# Early administration of high protein for critically ill patients with acute kidney injury?

**DOI:** 10.1186/s13054-023-04764-4

**Published:** 2023-12-07

**Authors:** Hasan M. Al-Dorzi, Yaseen M. Arabi

**Affiliations:** College of Medicine, King Saud Bin Abdulaziz University for Health Sciences and King Abdullah International Medical Research Center and Intensive Care Department, Ministry of National Guard-Health Affairs, Mail Code 1425, PO Box 22490, 11426 Riyadh, Saudi Arabia

During the acute phase of critical illness, patients experience a hypercatabolic state. It is generally accepted that the early catabolic phase (typically 4–7 days) is followed by a metabolically neutral phase followed by a recovery anabolic phase. Impairment of renal function has additional negative effects on protein metabolism. Renal replacement therapy triggers further protein loss, estimated in one study to vary between 5 and 19 g per day, being most pronounced with continuous renal replacement therapy. Higher protein intake has been proposed to mitigate these changes, and these changes collectively suggest that patients with acute kidney injury (AKI) require high protein intake. However, higher protein intake, especially during the acute phase of critical illness, may have detrimental effects, as it increases urea production, stimulates glucagon secretion, inhibits autophagy, and may negatively affect kidney function (Additional file 1: References 1–14, Online supplement).

Data supporting the optimal protein dose in patients with AKI are limited. An observational study in patients with AKI suggested that low caloric and protein intake were independently associated with hospital mortality. On the other hand, in a post hoc analysis of the RENAL (randomized evaluation of normal versus augmented level of replacement therapy) trial, there was no association between higher versus lower protein intake and mortality (Additional file 1: References 15, 16, Online supplement).

There is limited data from randomized clinical trials (RCTs) on the early administration of high protein in critically ill patients. In one RCT (*n* = 474), daily supplementation of up to 100 g of IV amino acids or standard care did not affect the primary outcome (mean duration of renal dysfunction). A post hoc analysis of this trial found that patients with normal kidney function at randomization who received IV amino acids had lower 90-day mortality, whereas there was no mortality benefit in the subgroup of patients with baseline kidney dysfunction. Another post hoc analysis of the REDOXS (REducing Deaths due to OXidative Stress) trial that compared glutamine with placebo in mechanically ventilated patients found worse outcomes with glutamine in patients with a baseline renal dysfunction, especially in the group that did not eventually receive hemodialysis. In the EPaNIC trial (Early Parenteral Nutrition Completing Enteral Nutrition in Adult Critically Ill Patients), adults in the intensive care unit (ICU) were randomized to early initiation of parenteral nutrition (within 48 h after ICU admission) to supplement enteral energy and protein intake or to late initiation of parenteral nutrition (after day 8). A predefined secondary analysis found that the timing of parenteral nutrition did not affect the incidence of AKI, but early initiation seemed to slow renal recovery in patients with stage 2 AKI. Early parenteral nutrition increased plasma urea, urea/creatinine ratio, and nitrogen excretion beginning on the first day of amino acid infusion (Additional file 1: References 17–21, Online supplement).

With the premise of mitigating the catabolic state, clinical practice guidelines have generally recommended the administration of higher protein intake in critically ill patients than in healthy individuals (World Health Organization recommendations: 0.7–0.8 g/kg/day) [1], although the recommended dose varies among different guidelines (Table 1). The 2021 European Society for Clinical Nutrition and Metabolism (ESPEN) clinical practice guidelines state that “protein prescription shall not be reduced in order to avoid or delay renal replacement therapy start in critically ill patients with AKI or chronic kidney disease” [2]. The ESPEN clinical practice guidelines recommend doses from 1.0 to 1.7 g/kg/day, based on pre-hospitalization body weight or usual body weight and depending on the use of renal replacement therapy [2]. The American Society for Parenteral and Enteral Nutrition/ Society of Critical Care Medicine (ASPEN/SCCM) clinical practice guidelines recommend 1.2–2.5 g/kg/day based on actual body weight in ICU patients with AKI [3].

Recently, the results of the EFFORT (The Effect of Higher Protein Dosing in Critically Ill Patients) trial were published. The trial compared higher (≥ 2.2 g/kg/day) versus lower (≤ 1.2 g/kg/day) protein dose in mechanically ventilated patients. The assigned protein dose was commenced as soon as possible after randomization and was achieved by enteral nutrition, parenteral nutrition, or both. The study found no improvement in the time-to-discharge-alive from the hospital with a higher protein dose [4]. Worse outcomes were observed in the pre-specified subgroup of patients with AKI [4]. Patients with AKI in the high protein group received 1.5 ± 0.5 g/kg/day versus 0.9 ± 0.3 g/kg/day in the usual protein group [5]. High protein was associated with a slower time-to-discharge alive from the hospital (hazard ratio 0.5, 95% CI 0.4–0.8) and higher 60-day mortality (relative risk 1.4, 95% CI 1.1–1.8) [5]. The harmful effect of higher protein seemed to occur mainly in patients who did not receive renal replacement therapy (*p* for interaction = 0.10 for both outcomes) [5]. On the other hand, protein dose was not associated with the incidence of AKI nor with the provision or duration of renal replacement therapy [5]. High protein intake resulted in a slight increase in levels of urea when compared to patients with the usual dose of protein (19.7 ± 9.8 vs. 17.6 ± 9.7 mmol/L; *p* = 0.04). As acknowledged by the authors, the study should be interpreted in the context of being based on exploratory subgroup analysis and having used post-randomization data (up to 7 days after ICU admission) to define the AKI subgroup. Nevertheless, the findings are clinically important and demonstrate that early administration of high protein in patients with AKI was not beneficial and might have been harmful in patients who were not receiving renal replacement therapy.

How protein should be managed after the acute phase? The answer to this question remains uncertain. Observational data suggest that lower earlier protein and higher later protein may be advantageous. Further data from ongoing clinical trials (REPLENISH trial [REPLacing Protein via Enteral Nutrition in a Stepwise ApproacH in critically ill patients], TARGET protein trial and PRECISE trial [PRotEin provision in Critical IllneSs]) will likely provide more insight into when and how much to give protein in critically ill patients in general, including those with AKI (Additional file 1: References 26–30, Online supplement).

**Table 1:** Current clinical practice guidelines on protein requirement in patients with acute kidney injury (AKI)

Guideline	Year	Patients	Recommendation	Quality of evidence
ASPEN/SCCM[3]	2016	ICU patients with AKI not on hemodialysis or CRRT	Follow the standard ICU recommendations for protein (1.2–2 g/kg actual BW per day)	Expert opinion^a^
		ICU patients on hemodialysis or CRRT	Increased protein, up to a maximum of 2.5 g/kg per day	Very low^a^
ESPEN[2]	2021	Hospitalized patient with AKI, AKI on CKD, CKD, with acute/ critical illness, not on KRT	Start with 1 g/kg BW per day, and gradually increase up to 1.3 g/kg BW per day if tolerated^b^	Grade of recommendation 0^a^
		Critically ill patients with AKI or AKI on CKD or CKD with KF on conventional intermittent KRT	1.3 to 1.5 g/kg BW per day^b^	Grade of recommendation 0^a^
		Critically ill patients with AKI or AKI on CKD or CKD with KF on CKRT or PIKRT	1.5 g/kg BW per day up to 1.7 g/kg BW per day^b^	Grade of recommendation 0, Consensus (82.6% agreement)^a^

^a^As defined by the authors of the guidelines

^b^Pre-hospitalization body weight or usual body weight may be preferred over the ideal body weight. Actual body weight should not be considered for a protein prescription, as per the ESPEN guidelines

AKI: acute kidney injury, BW: body weight, CKD: chronic kidney disease, KF: kidney failure, CRRT/CKRT: continuous renal/kideny replacement therapy, PIKRT: Prolonged Intermittent Kidney Replacement

### Supplementary Information

**Additional file 1.** Supplementary References: [1–26].

## Data Availability

Not applicable.
