# Supplementary material for: Early administration of high protein for critically ill patients with acute kidney injury?
Source: Crit Care. 2023 Dec 7;27:484. doi: 10.1186/s13054-023-04764-4 (PMC10704753; doi:10.1186/s13054-023-04764-4)
Supplement: Supplementary file 1 — Additional file 1. Supplementary References: [1–26]. [file 13054_2023_4764_MOESM1_ESM.docx]

**Early Administration of High Protein for Critically Ill Patients with Acute Kidney Injury?**

**Online Supplement**

**Supplementary References:** [1-26]

1. Puthucheary ZA, Rawal J, McPhail M, Connolly B, Ratnayake G, Chan P, Hopkinson NS, Phadke R, Dew T, Sidhu PS: **Acute skeletal muscle wasting in critical illness**. *Jama* 2013, **310**(15):1591-1600.

2. Berbel MN, Pinto MPR, Ponce D, Balbi AL: **Nutritional aspects in acute kidney injury**. *Revista da Associação Médica Brasileira (English Edition)* 2011, **57**(5):587-592.

3. Chima CS, Meyer L, Hummell AC, Bosworth C, Heyka R, Paganini E, Werynski A: **Protein catabolic rate in patients with acute renal failure on continuous arteriovenous hemofiltration and total parenteral nutrition**. *Journal of the American Society of Nephrology* 1993, **3**(8):1516-1521.

4. Fiaccadori E, Lombardi M, LEONARDI S, ROTELLI CF, TORTORELLA G, BORGHETTI A: **Prevalence and clinical outcome associated with preexisting malnutrition in acute renal failure: a prospective cohort study**. *Journal of the American Society of Nephrology* 1999, **10**(3):581-593.

5. Preiser JC, Ichai C, Orban JC, Groeneveld AB: **Metabolic response to the stress of critical illness**. *Br J Anaesth* 2014, **113**(6):945-954.

6. Preiser JC, Arabi YM, Berger MM, Casaer M, McClave S, Montejo-Gonzalez JC, Peake S, Reintam Blaser A, Van den Berghe G, van Zanten A *et al*: **A guide to enteral nutrition in intensive care units: 10 expert tips for the daily practice**. *Crit Care* 2021, **25**(1):424.

7. Reintam Blaser A, Berger MM: **Early or Late Feeding after ICU Admission?** *Nutrients* 2017, **9**(12).

8. Singer P, Blaser AR, Berger MM, Alhazzani W, Calder PC, Casaer MP, Hiesmayr M, Mayer K, Montejo JC, Pichard C: **ESPEN guideline on clinical nutrition in the intensive care unit**. *Clin Nutr* 2019, **38**(1):48-79.

9. Fiaccadori E, Sabatino A, Barazzoni R, Carrero JJ, Cupisti A, De Waele E, Jonckheer J, Singer P, Cuerda C: **ESPEN guideline on clinical nutrition in hospitalized patients with acute or chronic kidney disease**. *Clin Nutr* 2021, **40**(4):1644-1668.

10. Oh WC, Mafrici B, Rigby M, Harvey D, Sharman A, Allen JC, Mahajan R, Gardner DS, Devonald MAJ: **Micronutrient and Amino Acid Losses During Renal Replacement Therapy for Acute Kidney Injury**. *Kidney Int Rep* 2019, **4**(8):1094-1108.

11. Vanhorebeek I, Verbruggen S, Casaer MP, Gunst J, Wouters PJ, Hanot J, Guerra GG, Vlasselaers D, Joosten K, Van den Berghe G: **Effect of early supplemental parenteral nutrition in the paediatric ICU: a preplanned observational study of post-randomisation treatments in the PEPaNIC trial**. *The Lancet Respiratory Medicine* 2017, **5**(6):475-483.

12. Thiessen SE, Derde S, Derese I, Dufour T, Vega CA, Langouche L, Goossens C, Peersman N, Vermeersch P, Vander Perre S: **Role of glucagon in catabolism and muscle wasting of critical illness and modulation by nutrition**. *American journal of respiratory and critical care medicine* 2017, **196**(9):1131-1143.

13. Van Dyck L, Casaer MP, Gunst J: **Autophagy and its implications against early full nutrition support in critical illness**. *Nutrition in Clinical Practice* 2018, **33**(3):339-347.

14. Fouque D, Aparicio M: **Eleven reasons to control the protein intake of patients with chronic kidney disease**. *Nature clinical practice Nephrology* 2007, **3**(7):383-392.

15. Bufarah M, Costa N, Losilla M, Reis N, Silva M, Balbi A, Ponce D: **Low caloric and protein intake is associated with mortality in patients with acute kidney injury**. *Clinical nutrition ESPEN* 2018, **24**:66-70.

16. Bellomo R, Cass A, Cole L, Finfer S, Gallagher M, Lee J, Lo S, McArthur C, McGuinness S, Norton R: **Daily protein intake and patient outcomes in severe acute kidney injury: findings of the randomized evaluation of normal versus augmented level of replacement therapy (RENAL) trial**. *Blood purification* 2014, **37**(4):325-334.

17. Doig GS, Simpson F, Bellomo R, Heighes PT, Sweetman EA, Chesher D, Pollock C, Davies A, Botha J, Harrigan P *et al*: **Intravenous amino acid therapy for kidney function in critically ill patients: a randomized controlled trial**. *Intensive Care Med* 2015, **41**(7):1197-1208.

18. Zhu R, Allingstrup MJ, Perner A, Doig GS: **The effect of IV amino acid supplementation on mortality in ICU patients may be dependent on kidney function: post hoc subgroup analyses of a multicenter randomized trial**. *Critical Care Medicine* 2018, **46**(8):1293-1301.

19. Heyland DK, Elke G, Cook D, Berger MM, Wischmeyer PE, Albert M, Muscedere J, Jones G, Day AG, Group CCCT: **Glutamine and antioxidants in the critically ill patient: a post hoc analysis of a large‐scale randomized trial**. *Journal of Parenteral and Enteral Nutrition* 2015, **39**(4):401-409.

20. Casaer MP, Mesotten D, Hermans G, Wouters PJ, Schetz M, Meyfroidt G, Van Cromphaut S, Ingels C, Meersseman P, Muller J *et al*: **Early versus late parenteral nutrition in critically ill adults**. *N Engl J Med* 2011, **365**(6):506-517.

21. Gunst J, Vanhorebeek I, Casaer MP, Hermans G, Wouters PJ, Dubois J, Claes K, Schetz M, Van den Berghe G: **Impact of early parenteral nutrition on metabolism and kidney injury**. *J Am Soc Nephrol* 2013, **24**(6):995-1005.

22. Koekkoek W, van Setten CHC, Olthof LE, Kars J, van Zanten ARH: **Timing of PROTein INtake and clinical outcomes of adult critically ill patients on prolonged mechanical VENTilation: The PROTINVENT retrospective study**. *Clin Nutr* 2019, **38**(2):883-890.

23. de Koning MLY, Koekkoek W, Kars J, van Zanten ARH: **Association of PROtein and CAloric Intake and Clinical Outcomes in Adult SEPTic and Non-Septic ICU Patients on Prolonged Mechanical Ventilation: The PROCASEPT Retrospective Study**. *JPEN J Parenter Enteral Nutr* 2020, **44**(3):434-443.

24. Summers MJ, Lee-anne SC, Bellomo R, Chapman MJ, Ferrie S, Finnis ME, French C, Hurford S, Kakho N, Karahalios A: **Study protocol for TARGET protein: The effect of augmented administration of enteral protein to critically ill adults on clinical outcomes: A cluster randomised, cross-sectional, double cross-over, clinical trial**. *Critical Care and Resuscitation* 2023.

25. Arabi YM, Al-Dorzi HM, Sadat M, Muharib D, Algethamy H, Al-Hameed F, Mady A, AlGhamdi A, Almekhlafi GA, Al-Fares AA: **Replacing protein via enteral nutrition in a stepwise approach in critically ill patients: the REPLENISH randomized clinical trial protocol**. *Trials* 2023, **24**(1):485.

26. van Gassel RJ, Bels JL, Tartaglia K, van Bussel BC, van Kuijk SM, Deane AM, Puthucheary Z, Weijs PJ, Vloet L, Beishuizen B: **The impact of high versus standard enteral protein provision on functional recovery following intensive care admission (PRECISE trial): study protocol for a randomized controlled, quadruple blinded, multicenter, parallel group trial in mechanically ventilated patients**. *Trials* 2023, **24**(1):416.
